# Prognostic Value of Glycated Hemoglobin in Frail Older Diabetic Patients With Hip Fracture

**DOI:** 10.3389/fendo.2021.770400

**Published:** 2021-11-18

**Authors:** Simone Paterni, Chukwuma Okoye, Alessia M. Calabrese, Filippo Niccolai, Antonio Polini, Nadia Caraccio, Valeria Calsolaro, Fabio Monzani

**Affiliations:** Geriatrics Unit, Department of Clinical and Experimental Medicine, University of Pisa, Pisa, Italy

**Keywords:** T2DM, older patient, glycated hemoglobin, hip fracture, HbA1c, frailty

## Abstract

**Background:**

Previous studies have shown increased risk of fracture in older patients with poor or strict glycemic control (glycated hemoglobin, HbA1c, ≥ 8% or < 6-7% respectively); however, these reports did not investigate the oldest-old population. Comprehensive geriatric assessment (CGA) and a patient-centered approach have been proven to improve the quality of care in the management of Type 2 Diabetes Mellitus (T2DM) in the older patients, but data regarding T2DM in patients with fragility fractures are still lacking.

**Aim:**

To investigate the prognostic role of HbA1c and frailty level in older diabetic patients admitted for hip fracture.

**Methods:**

Prospective observational cohort study conducted on diabetic geriatric patients consecutively hospitalized for hip fracture in the orthogeriatric unit of a tertiary care hospital. Preoperative comprehensive geriatric assessment (CGA) was performed. Using the Clinical Frailty Scale (CFS), diabetic patients were categorized in robust (CFS < 5) and frail (CFS ≥ 5), and further stratified according to HbA1c values [Tertile 1 (T1) HbA1c < 48 mmol/mol, Tertile 2 (T2) 48-58 mmol/mol and Tertile 3 (T3) > 58 mmol/mol). Comparisons between continuous variables were performed with analysis of non-parametric test for independent samples, while relationships between categorical variables were assessed by chi-square test. Using logistic multivariate regression, we evaluated the determinants of 1-year all-cause mortality in diabetic older patients with hip fracture.

**Results:**

Among the 1319 older patients (mean age 82.8 ± 7.5 years, 75.9% females) hospitalized for hip fracture, 204 (15.5%) had a previous diagnosis of T2DM. T2DM patients showed an increased proportion of multiple concurrent fractures occurred during the accidental fall or syncope (12.7% *vs* 11.2%, p=0.02). One-year mortality after hip fracture surgery was significantly higher in T2DM as compared to not diabetic patients (21.2% *vs* 12.5%, p<0.001). No significant difference in mortality was found across HbA1c tertiles; however, frail diabetic patients in the second and third HbA1c tertiles showed higher mortality risk compared to the robust counterparts (26.9% *vs* 5%, p=0.001 for T2 and 43.5% *vs* 13.3%, p=<0.05 for T3), while no difference was observed among those in T1.

**Conclusions:**

Frail patients with HbA1c ≥ 48 mmol/L showed an increased mortality risk as compared to robust counterparts. CFS represents an important tool to select diabetic subjects with higher likelihood of adverse outcome.

## Introduction

The older population is strongly affected by type 2 diabetes mellitus (T2DM), with an estimated prevalence of 19.3% and 135.6 million cases worldwide (1). T2DM is associated with an increased risk of major fracture; in particular, the risk of hip fracture is 20-30% higher in diabetic patients compared to not diabetic ones (2, 3). Furthermore, post-hip fracture mortality is higher in T2DM patients, with an increased risk of death up to 44% in the first year after the event (3). While the contribution of T2DM to low bone density is still debated, it is recognized that the longer the disease duration, the higher the trabecular bone microarchitecture and mechanical properties damage (4). Moreover, the combination of both T2DM-related intrinsic (i.e., vision impairment and loss of balance due to neuropathy and retinopathy complications) and extrinsic factors (for example, treatment-induced hypoglycemia), confers to the diabetic older patient an increased likelihood of falls, which are well-known to be associated with poor outcomes in advanced age (5–7). Given that, targeting research toward modifiable risk factor is pivotal for this class of frail patients. The role of glycometabolic control, usually expressed in terms of glycated hemoglobin (HbA1c), in bone homeostasis is controversial. HbA1c is a reliable indicator of long-term glycemic control, providing a picture of patients’ average blood glucose levels during the previous two to three months (8). The formation of HbA1c inside erythrocytes is the best-known example of non-enzymatic protein glycation, which consists in the addition of a free carbonyl group of a reducing sugar to the free amino groups of proteins, resulting in the formation of early and advanced glycation end-products (AGEs). AGEs may be synthetized either rapidly or over long times induced by several systemic pathways, thereby accounting for their roles in multiple settings and disease states (9–12). Previous studies have shown an increased risk of fracture in older patients with either poor (HbA1c ≥ 8%) or strict glycemic control (HbA1c < 6-7%); however, these reports did not seek to investigate the oldest-old population (13–16). Indeed, the comprehensive geriatric assessment (CGA) and a patient-centered approach have been proven to improve the quality of care in the management of T2DM in the older patients, but data regarding T2DM and orthogeriatric patients are still lacking (17, 18). The geriatric individual with T2DM represent the phenotype of the so-called “complex patient”, where a multi-dimensional assessment, considering social, behavioral and clinical evaluation, is necessary to improve outcomes and quality of life. In particular, frailty assessment is a key-element to tailor T2DM medication in the older patients, and several scientific societies have proposed different approaches in terms of glycometabolic control in older people and in frail patients (18, 19). However, as regards the older population with hip fracture, the paucity of studies about glycometabolic control and frailty status makes it difficult to draw meaningful conclusions. Several studies showed a significant relationship between glycemic control and increased risk of hip fracture in older diabetic patients; however, none of these considered the degree of frailty (14, 20). On the other hand, Li et al. reported a higher fracturative risk in frail diabetic patients, without considering the glycometabolic control (21). For decades, the orthogeriatric method allowed to select complex patients hospitalized for fragility fracture, with the aim of promoting their clinical stabilization through a tailored approach, centered on the individual, with positive impact in terms of recovery and survival (22). The identification of key elements in order to stratify fractured patients and to assign the correct degree of assistance, especially in condition of high care burden, has a potentially positive value in optimizing the orthogeriatric method.

The aim of the study was to investigate the relationship between glycated hemoglobin and frailty status in orthogeriatric patients. Secondary endpoint was to define the usefulness of CGA, with its many components, in the diabetic patient with hip fracture in the orthogeriatric setting.

## Materials and Methods

A prospective observational cohort study was conducted evaluating diabetic geriatric patients hospitalized for hip fracture in the Orthopedic Traumatology Unit of a tertiary care hospital and included in our orthogeriatric service, between April 2013 and December 2018. The study complied with the Declaration of Helsinki and was approved by the local Ethic Committee. The patients/participants provided their written informed consent to participate in this study; the legally authorized delegate provided informed consent in case of patients who were temporarily or permanently without capacity. Demographic characteristic and clinical history were recorded. Preoperative comprehensive geriatric assessment (CGA) was performed, including: cognitive evaluation using the Short Portable Mental Status Questionnaire (SPMSQ) (23), level of autonomy in terms of independence in the performance of basic (ADL) (24), and instrumental (IADL) (25), activities of daily living, risk of malnutrition through the Mini Nutritional Assessment-Short Form (MNA-SF) (26). Comorbidity was assessed with the Cumulative Illness Rating Scale (CIRS-c) (27). Information about the presence of hyperactive delirium in the perioperative period was also reported. The level of fitness or frailty was described using the Clinical Frailty Scale (CFS) (28). We collected data on total numbers of drugs and antidiabetic therapy (diet, metformin, sulfonylureas, pioglitazone, repaglinide, acarbose, DPP4-inhibitors, GLP-1R agonists, gliflozins, insulins). Data regarding history of hypoglycemic episodes were collected from inpatients assuming hypoglycemia-inducing drugs (HD) such as sulfonylureas, repaglinide and rapid-acting insulin. We also recorded information about vision and hearing impairments. Type of fall (accidental, syncope), surgical procedure (internal fixation with intramedullary nail, replacement with prosthesis), numbers of previous and concurrent fractures were also specified. Baseline value of HbA1c was recorded. Diabetic patients were divided into three sub-groups according to the HbA1c values [Tertile 1 (T1) HbA1c < 48 mmol/mol, Tertile 2 (T2) 48-58 mmol/mol and Tertile 3 (T3) > 58 mmol/mol); patients were further categorized as frail and robust on the basis of CFS (respectively, ≥ 5 and < 5). Survival during follow-up was assessed by a regional administrative archive that records death of any hospitalized patients living in Tuscany.

### Statistical Analysis

Statistical analysis was performed with IBM SPSS Statistic (IBM SPSS Statistic version 27.0 lnk IBM Corporation and its licensor 1989-2020). Continuous variables were presented as mean ± standard deviation, ordinal variables as median and interquartile range (IQR), and categorical variables as percentage. Mann-Whitney and chi-square test were used for multiple comparisons. Multivariate logistic regression analysis was performed to identify factors associated with one-year all-cause mortality. Backward stepwise multivariate logistic regression was performed with the following continuous and categorical covariates: age, sex, CIRS-c, CFS, HbA1c, number of concurrent fractures, previous fractures, and type of surgical procedure. Probability for removal of variables in the model was set at P = .10 or higher. Estimate odds ratios (ORs) with 95% confidence intervals (CIs) were obtained. Tests were performed considering a level of significance of 5%.

## Results

Among the 1319 geriatric patients with hip fracture enrolled in the study (mean age 82.8 ± 7.5 years, 75.9% females), 204 (15.5%) had a previous diagnosis of T2DM. As reported in **Table 1**, T2DM patients were less frequently females (69.6% *vs* 77.0%, p=0.02) and showed a higher burden of comorbidities [CIRS-c (± SD) 3.7 ± 1.6 *vs* 3 ± 1.6, p< 0.0001] than the counterpart. No significant differences were detected in terms of disabilities [median (IQR), ADL: 5(3) *vs* 5(3), p = 0.84; IADL: 4(7) *vs* 4(6), p = 0.92], cognitive impairment [median (IQR), SPMSQ: 1(4) *vs* 2 (4), p = 0.37], and prevalence of malnutrition (18.6% *vs* 14.3%, p=0.14). T2DM patients had more frequently concomitant fractures occurred during the accidental fall or syncope [26(12.7%) *vs* 125(11.2%), p = 0.02) and a higher, not significant, prevalence of previous fractures [81(39.7%) *vs* 396(35.5%), p = 0.23]. Diabetics patients faced longer hospital stay [7.3 ± 2.7 *vs* 6.1 ± 2.7 days, p<0.0001], and an increased number of prescribed drugs (mean 6.8 ± 2.9 *vs* 4.1 ± 3.1, p< 0.0001) than the counterpart. Nonetheless, a not significantly lower incidence of delirium [17(8.3%) *vs* 112(10%), p = 0.44] was observed among T2DM patients.

**Table 1:** Clinical and demographic characteristics of diabetic and non-diabetic patients with hip fracture hospitalized in our orthogeriatric ward.

	Whole cohort (*n* = 1319)	Diabetics (*n* = 204)	Not diabetics (*n* = 1115)	*p*
**Gender** (F, %)	1001 (75.9)	142 (69.6)	859 (77)	0.02
**Age** (mean ± SD)	82.8 ± 7.5	81.8 ± 7.7	82.9 ± 7.4	0.68
**Length of stay** (days)	6.3 ± 2.8	7.3 ± 2.7	6.1 ± 2.7	<0.0001
**ADL** (median, IQR)	5 (3)	5 (3)	5 (3)	0.84
**IADL** (median, IQR)	4 (6)	4 (7)	4 (6)	0.92
**SPMSQ** (median, IQR)	2 (4)	1 (4)	2 (4)	0.37
**MNA** (malnourished or at risk of malnutrition, %)	198 (15)	38 (18.6)	160 (14.3)	0.14
**CIRS-C**	3.1 ± 1.6	3.7 ± 1.6	3 ± 1.6	<0.0001
**Delirium** (%)	129(9.8)	17(8.3)	112 (10)	0.44
**Amount of medications**	4.5 ± 3.2	6.8 ± 2.9	4.1 ± 3.1	<0.0001
**More than one concurrent fracture** (%)	151(11.4)	26 (12.7)	125 (11.2)	0.02
**Previous fractures** (%)	477 (36.2)	81 (39.7)	396 (35.5)	0.23

ADL, Activities of Daily Living; IADL, Instrumental Activities of Daily Living; SPMSQ, Short Portable Mental Status Questionnaire; MNA, Mini- Nutritional Assessment; CIRS-C, Cumulative Illness Rating Scale-Comorbidity.

As shown in **Table 2**, diabetic patients were split in tertiles according to HbA1c values [T1 = 91(44.6%), T2 = 65(31.9%), T3 = 48(23.5%)], and further categorized in “robust” and “frail” (**Table 3**).

**Table 2:** Clinical and demographic characteristics of diabetic orthogeriatric patients categorized in tertiles of glycated hemoglobin.

		Whole diabetic cohort (*n* = 204)	T1 (HbA1c<48 mmol/mol) (*n* = 91)	T2 (HbA1c 48-58 mmol/mol) (*n* = 65)	T3 (HbA1c > 58 mmol/mol) (*n* = 48)	*p*
**Women** (%)	142 (69.6)		57 (62.6)	46 (70.8)	39 (81.2)	0.12
**Age** (mean ± SD)	81.8 ± 7.7		81.0 ± 8.5	81.5 ± 6.8	83.7 ± 7.3	0.37
**ADL** (median, IQR)	5 (3)		5 (3)	5 (3)	5 (4)	0.21
**IADL** (median, IQR)	4 (6)		4 (6)	5 (6)	3 (5)	0.08
**SPMSQ** (median, IQR)	1 (6)		1 (4)	1 (3.5)	3 (5)	0.08
**Robust** (CFS ≤ 5, %)	98 (48.1)		44 (48.3)	35 (53.8)	19 (39.6)	0.29
**MNA** (malnourished or at risk, %)	35 (17.2)		18 (19.8)	11 (16.9)	6 (12.5)	0.54
**CIRS-C**	3.6 ± 1.6		3.8 ± 1.7	3.3 ± 1.5	3.8 ± 1.6	0.67
**Arterial hypertension** (%)	141 (69.1)		65 (71.4)	47 (72.3)	29 (60.4)	0.326
**CAD** (%)	20 (9.8)		13 (14.3)	1 (1.5)	6 (12.5)	0.024
**AF** (%)	37 (18.2)		17 (18.7)	10 (15.6)	10 (20.8)	0.770
**CKD** (%)	47 (23)		25 (27.5)	11 (16.9)	11 (22.9)	0.304
**COPD** (%)	20 (9.8)		11 (12.1)	6 (9.2)	3 (6.3)	0.536
**Delirium** (%)	17(8.3)		6 (6.6)	4 (6.1)	7 (14.6)	0.23
**Type of falls** (%):						
accidental fall	194 (95.1),		83 (91.2),	64 (98.5),	47 (97.9),	0.07
syncope	10 (4.9)		8 (8.8)	1 (1.5)	1 (2.1)	
**More than one concurrent fracture** (%)	26 (12.7)		13 (14.3)	9 (13.8)	4 (8.3)	0.73

ADL, Activities of Daily Living; IADL, Instrumental Activities of Daily Living; SPMSQ, Short Portable Mental Status Questionnaire; CFS, Clinical Frailty Scale; MNA, Mini-Nutritional Assessment; CIRS-C, Cumulative Illness Rating Scale-Comorbidity; CAD, Coronary Artery Disease; AF, Atrial Fibrillation; CKD, Chronic Kidney Disease; COPD, Chronic Obstructive Pulmonary Disease.

**Table 3:** Antidiabetic therapy in orthogeriatric patients according to frailty degree.

	Whole cohort (*n* = 204)	Robust (CFS< 5) (*n* = 101)	Frail (CFS >= 5) (*n* = 103)	*p*
**Rapid-acting insulin** (%)	38 (18.9)	25 (25.5)	13 (12.6)	0.04
**Long-acting insulin** (%)	41 (20.4)	27 (27.5)	14 (13.6)	0.04
**Oral antidiabetics** (%)	133 (66.2)	72 (73.5)	61 (59.2)	0.03
**Dietetic therapy** (%)	37(18.4)	18 (18.4)	19 (18.4)	0.08
**Metformin** (%)	109 (54.2)	57 (58.2)	52 (50.5)	0.27
**Sulfonylureas** (%)	18 (8.9)	10 (10.2)	8 (7.8)	0.54
**Pioglitazone** (%)	2 (1)	1 (1)	1 (1)	0.97
**Repaglinide** (%)	18 (8.9)	7 (7.1)	11 (10.7)	0.39
**Acarbose** (%)	1 (0.5)	0	1 (1)	0.33
**DPPIV*i*** (%)	28 (13.8)	18 (18.4)	10 (9.7)	0.08
**GLP1R*a*** (%)	2 (1)	1 (1)	1 (1)	0.97
**Gliflozins** (%)	4 (2)	4 (4.1)	0	0.04

DPP-IV-I, Dipeptidyl peptidase-4 inhibitor; GLP1R-a, Glucagon-like peptide-1 receptor agonist.

Across the tertiles of HbA1c, no significant differences were found in terms of age (83.7 ± 7.3, *vs* 81.0 ± 8.5 and 81.5 ± 6.8 years, respectively, p=0.37) and gender proportion [females: 39(81.2%) *vs* 57(62.6%) and 46(70.8%) respectively, p = 0.12]. According to CFS, patients in T3 were less frequently “robust”, compared to those in T1 and T2 [19(39.6%) *vs* 44(48.3%) and 35(53.9%), p=0.29], although less frequently malnourished (12.5% *vs* 19.8% and 16.9% respectively, p = 0.54). As shown in **Table 2**, patients in T2 had the lowest prevalence of coronary artery disease compared to T1 and T3 (respectively, 1.5% *vs* 14.3% *vs* 12.5%, p = 0.024), while no statistical differences were found between the tertiles of HbA1c in terms of other comorbidities. Finally, patients in T3 experienced more frequently delirium during the hospital stay than T1 and T2 peers, (respectively, 14.6% *vs* 6.6% and 6.1%, p = 0.23).

Patients in the first tertile of HbA1c (<48 mmol/mol) showed a higher prevalence of syncopal falls compared to those in T2 and T3 (8.8% *vs* 1.5% and 2.1% respectively, p=0.05). Out of 73 patients assuming HD, 45 (61.6%) of them had experienced at least one symptomatic hypoglycemic episode. Frail T2DM patients less frequently received both basal and rapid-acting insulin therapy [respectively, 14(13.6%) *vs* 27(27.5%), p = 0.04 and 13 (12.6%) *vs* 25(25.5%), p = 0.04], as well as oral antidiabetic treatment [61(59.2%) *vs* 72(73.5%), p = 0.03] compared to robust ones. More than half of the diabetic population received metformin (54.2%) with no statistical differences between robust and frail patients [57(58.2%) *vs* 52(50.5%), p = 0.27]. There were no differences in the prescription of sulfonylureas [10(10.2%) *vs* 8(7.8%), p = 0.54], pioglitazone [1(1%) *vs* 1(1%), p = 0.97], repaglinide [7(7.1% *vs* 11 (10.7%), p = 0.39], acarbose [0 *vs* 1 (1%), p = 0.33], dipeptidyl peptidase-4 inhibitors [18(18.4%) *vs* 10(9.7%), p = 0.08] and glucagon like peptid-1 receptor agonist [1(1%) *vs* 1 (1%), p = 0.97], while gliflozins resulted more prescribed in robust patients [4(4.1%) *vs* 0, p = 0.04]. No statistically significant differences were found in dietetic treatment between robust and frail patients [18(18.4%) *vs* 19(18.4%), p = 0.08].

**Figure 1:**
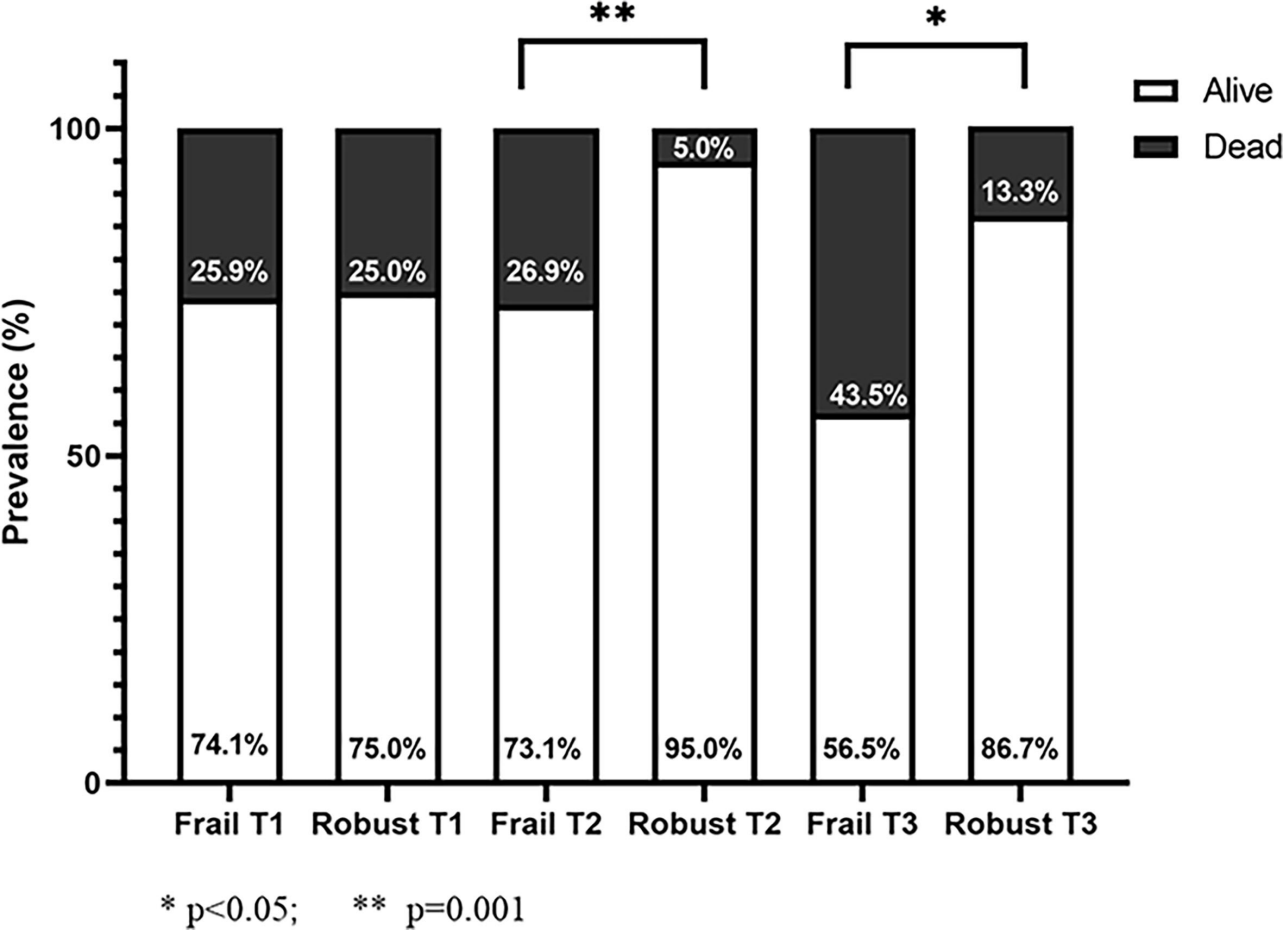
Mortality rate of diabetic patients stratified by frailty degree (frail: CFS ≥ 5; robust: CFS < 5) according to HbA1c tertiles.

The prevalence of one-year mortality after hip fractures surgery was significantly higher among T2DM patients as compared to the counterpart (21% and 12.4% respectively, p<0.001). Across the tertiles of HbA1c, patients in T3 had higher, although not statistically significant, one-year mortality (T3 = 29.8%, T1 = 21.1%, T2 = 16.1% p = 0.23]. In the whole cohort, frail T2DM patients showed a significantly increased mortality rate as compared to robust ones [31(30.4%) *vs* 10(10.5%), p = 0.001]. However, after stratifying patients according to HbA1c tertiles, no difference in mortality rate was found between robust and frail patients with strict glycemic control (HbA1c < 48 mmol/mol), while frail patients with not optimal glycemic control (HbA1c > 48 mmol/mol) showed a four-five times higher mortality compared to robust ones [T2: 26.9% *vs* 5% (p <0.001), T3: 43.5% *vs* 13.3% (p<0.05)] (**Figure 1**). The relationship between patients’ complexity and poor outcome was also confirmed by Multivariate Logistic analysis, were age and frailty degree emerged as the only two independent predictors of mortality (adjusted OR = 1.09 CI95% 1.03-1.15 and adjusted OR = 1.62 CI95% 1.25-2.01 respectively), as shown in **Supplemental Table 1**.

## Discussion

In this single-center prospective study, we evaluated a cohort of older patients admitted to a tertiary care hospital for hip fracture, receiving orthogeriatric care. It is well established that older patients with hip fracture, especially if frail, have a worse prognosis after surgery in the first year after discharge (29). Our study shows one-year prognostically negative impact of T2DM in older diabetic patients with a fractured femur (21.5% *vs* 12.5%) compared to non-diabetic patients, consistent with the literature (30). In our sample, the prevalence of T2DM was 15.5%, almost comparable to the national data for adults over 65s (16.5%) (31). Mean age was comparable to previous reports, defining an older study population in a range between 75 and 90 years, when the prevalence of osteoporosis and risk of falls and low-energy trauma is higher (22, 32). The prognosis of the whole cohort of diabetic patients with hip fracture was independent from the HbA1c levels measured at admission. Conflicting results have been reported regarding the possible association between high levels of HbA1c at admission and increased mortality after surgery on hip fracture or other orthopedic procedures; Puar et al. found an association between tight glycemic control (HbA1c < 7%) and greater risk of hip fracture in individuals being treated for type 2 diabetes mellitus (13). Gulcelik et al. showed that high HbA1c level was predictor of mortality in diabetic patients, together with advanced age and postoperative complications (17). On the contrary, Thörling et al. found that elevated HbA1c at admission was not associated with increased complications or mortality after hip fracture surgery (33). Noteworthy, after stratifying patients according to frailty status we demonstrated a huge elevation of mortality risk in frail patients with HbA1c > 48 mmol/L (Tertile 2 and 3). As shown in **Figure 1**, at equal HbA1c tertile, frail patients in T2 and T3 had respectively a 5 and 3 - times higher mortality compared to robust peers. Conversely, in T1, frailty status seems to not interfere with the mortality; this is not surprising since it is well recognized the detrimental prognostic impact of a strict glycemic control in older patients, in agreement with recent guidelines (34). Furthermore, at multivariate logistic analysis, age and frailty were identified as only independent predictors of mortality. Altogether, these findings highlight the importance of a patient-centered therapeutic approach, confirming that HbA1c *per se* is not a predictor of adverse outcomes, but rather an effect-modifier in specific clinical phenotypes. On the other hand, frailty seems to represent a crucial aspect that need to be considered when approaching a diabetic patient with hip fracture. On this regard, the Clinical Frailty Scale is a widely used and easy-to-apply tool for identifying frail geriatric patients, and has a high predictive value of negative outcomes and mortality in hip fractured patients (35, 36).

The link between frailty and diabetic disease has also been extensively described, both representing a negative prognostic feature (37, 38). In our study, older diabetic patients resulted severely affected by a greater burden of comorbidities and more frequently exposed to polypharmacy, defining a higher individual complexity than non-diabetic peers, which probably are the main causes of the longer hospitalization observed (39, 40). Despite what could be expected, there were no significant differences between diabetic and non-diabetic patients before hip fracture in terms of disability, in line with literature; neither the degree of cognitive impairment and the risk of malnutrition did differ between the two groups, as already reported (40). Although T2DM has been described as an independent risk factor for in-hospital delirium, in our study diabetic patients did not show more episodes of delirium than non-diabetic ones, with an overall prevalence lower than described in literature (41, 42). This counter-trend data may be explained by the smallness of the sample and the little difference in terms of cognitive impairment between diabetics and non-diabetics examined (43). Confirming a fracture propensity in diabetic patients, the coexistence of other fractures at admission was more common among diabetics, possibly due to the greater bone fragility associated with type 2 diabetes mellitus, and to the increased number of falls in the diabetic patient (44). The significantly higher number of concomitant fractures reported in patients with lower levels of HbA1c, who experienced more frequently syncopal falls, could be explained by the known association between falls and hypoglycemia. Moreover, 61.6% of patients receiving rapid-acting insulin and insulin secretagogues had history of at least one symptomatic hypoglycemic episode. This data confirms the need of a tailored approach in the frail older patient, who requires greater tolerance in terms of therapeutic target for glycometabolic control, especially if the use of drugs with high risk of hypoglycemia is inevitable (39, 41, 42). Interestingly, the CGA showed no significant differences in comorbidity and number of drugs, risk of malnutrition, length of hospitalization, onset of delirium and cognitive impairment. However, even though not significantly, it should be noted that diabetic patients with worse glycometabolic control (HbA1c > 58 mmol/mol) had worse scores on the Pfeiffer test, indicating a trend to cognitive decline, as well as a higher degree of disability in instrumental and basic daily functions. In this regards, worse glycometabolic control is associated with worse functional status and greater disability in IADL (33, 45) and, although T2DM is certainly associated with higher risk of cognitive dysfunction, the correlation with HbA1c levels has not been confirmed so far (46, 47). No differences were also found, across the three groups of different level of glycometabolic control, in the number of previous fracture events, nor for frequency of multiple fractures at admission. These data do not suggest a clear link between glycometabolic control and fracture risk, confirming the controversial role of HbA1c levels on this front (43, 44, 48, 49). Evaluating the antidiabetic drugs prescription in T2DM individuals, we have noted that two-third of patients were oral antidiabetic users, whereas one-third received insulin. Interestingly, robust patients received almost twice the antidiabetic treatment in comparison with non-robust peers, underlying a worrisome frailty-related drug under-prescription. Bearing in mind the huge increase of mortality risk observed in our cohort of frail patients with not optimal glycemic control, this finding appears particularly valuable. On the other hand, frail individuals more frequently received the hypoglycemic drug repaglinide as compared to robust ones, thus increasing the risk of accidental falls.

Being a single center study could be acknowledged as a limitation; certainly, further studies, multicenter and with larger cohorts, would help in confirming the role of frailty and glycemic control in the oldest patients admitted for hip fracture.

In conclusion, T2DM determines a greater complexity in older patients with hip fracture in terms of comorbidities and entails the need for longer hospitalizations and greater intensity of care. Our data confirm that the overlap of an acute systemic pathology, such as hip fracture, to the diabetes-frailty dyad causes a further worsening of the 1-year-prognosis after orthopedic surgery, specifically in frail patients. Frailty emerged as an independent mortality predictor for individuals with HbA1c levels higher than 48 mmol/mol; conversely, in patients with a stringent glycemic control, no differences were found in terms of mortality between frail and robust patients, highlighting the detrimental effect of hypoglycemia in the older patient regardless clinical status.

The routinary use of CGA and CFS in the clinical workup would improve the selection of subjects with greater frailty and disability, even among diabetics, in order to promote functional recovery and maintenance of homeostasis in the months following the fracture event. Specifically, the concomitant presence of frailty and non-optimal glycemic control at hospital admittance may allow the clinician to select patients with higher likelihood of adverse outcome.

## Data Availability Statement

The raw data supporting the conclusions of this article will be made available by the authors, without undue reservation.

## Ethics Statement

The studies involving human participants were reviewed and approved by Comitato etico regionale per la sperimentazione clinica della regione Toscana Sezione Area Vasta Nord Ovest Stabilimento Santa Chiara, Pisa. The patients/participants provided their written informed consent to participate in this study.

## Author Contributions

SP, CO, and FM conceived and designed the manuscript. SP and AC collected the data, reviewed the literature and prepared the draft of the manuscript. CO and AC analyzed the data. FN, AP, and NC supervised the literature search and revision. CO, VC, and FM supervised the literature search, reviewed and edited the manuscript. All authors contributed to the article and approved the submitted version.

## Conflict of Interest

The authors declare that the research was conducted in the absence of any commercial or financial relationships that could be construed as a potential conflict of interest.

## Publisher’s Note

All claims expressed in this article are solely those of the authors and do not necessarily represent those of their affiliated organizations, or those of the publisher, the editors and the reviewers. Any product that may be evaluated in this article, or claim that may be made by its manufacturer, is not guaranteed or endorsed by the publisher.
