# Supplementary material for: Prognostic Value of Glycated Hemoglobin in Frail Older Diabetic Patients With Hip Fracture
Source: Front Endocrinol (Lausanne). 2021 Nov 18;12:770400. doi: 10.3389/fendo.2021.770400 (PMC8637116; doi:10.3389/fendo.2021.770400)
Supplement: Supplementary file 1 [file Table_1.docx]

**Supplemental Table 1.** Multivariate Backward Stepwise Analysis

|  | **Univariate** | | **Multivariate 1-Step 7** | |
| --- | --- | --- | --- | --- |
| **Variable** | OR (95% CI) | β | OR (95% CI) | β |
| **Age** | 1.09 (1.03-1.16) | 0.089 | 1.09 (1.03-1.15) | 0.085 |
| **Sex** | 1.38 (0.54-3.55) | 0.324 |  |  |
| **CIRS-C** | 1.19 (0.92-1.55) | 0.180 |  |  |
| **CFS** | 1.59 (1.23-2.07) | 0.467 | 1.62 (1.25-2.01) | 0.481 |
| **HbA1c** | 1.01 (0.98-1.04) | 0.009 |  |  |
| **Number of concurrent fractures** | 1.22 (0.49-3.01) | 0.198 |  |  |
| **Previous fractures** | 1.49 (0.66-3.36) | 0.398 |  |  |
| **Type of surgical procedure** | 2.08 (0.86-5.03) | 0.734 |  |  |

CIRS-C: Cumulative Illness Rating Scale-Comorbidity; CFS: Clinical Frailty Scale; HbA1c: glycated hemoglobin
