# Supplementary material for: Prognostic Value of Glycated Hemoglobin in Frail Older Diabetic Patients With Hip Fracture
Source: Front Endocrinol (Lausanne). 2021 Nov 18;12:770400. doi: 10.3389/fendo.2021.770400 (PMC8637116; doi:10.3389/fendo.2021.770400)
Supplement: Supplementary file 2 [file Table_2.docx]

**Supplemental Table 2.** Metabolic characteristics of frail and robust patients stratified by tertiles of HbA1c

|  | **Pre-surgery characteristics** | **Robust** | **Frail** | **p** |
| --- | --- | --- | --- | --- |
|  | **Creatinine** (mg/dL) | 1.21 ± 1.2 | 1.27 ± 0.9 | 0.17 |
| **T1** (HbA1c <48 mmol/mol) | **Hemoglobin** (g/dL) | 11.4 ± 1.9 | 11.2 ± 1.8 | 0.75 |
|  | **Albumin** (g/dL) | 3.5 ± 0.5 | 3.4 ± 0.4 | 0.24 |
|  | **BMI** (kg/m^2^) | 26.5 ± 4.2 | 25.8 ± 4.4 | 0.66 |

|  | **Creatinine** (mg/dL) | 0.89 ± 0.3 | 1.04 ± 0.3 | 0.07 |
| --- | --- | --- | --- | --- |
| **T2** (HbA1c 48 - 58 mmol/mol) | **Hemoglobin** (g/dL) | 11.8 ± 1.8 | 11.3 ± 1.6 | 0.25 |
|  | **Albumin** (g/dL) | 3.6 ± 0.4 | 3.6 ± 0.4 | 0.85 |
|  | **BMI** (kg/m^2^) | 27.1 ± 4 | 24.2 ± 4 | 0.04 |

|  | **Creatinine** (mg/dL) | 0.93 ± 0.3 | 1.11 ± 0.6 | 0.65 |
| --- | --- | --- | --- | --- |
| **T3** (HbA1c > 58 mmol/mol) | **Hemoglobin** (g/dL) | 11.4 ± 1.5 | 11.3 ± 1.8 | 0.72 |
|  | **Albumin** (g/dL) | 3.6 ± 0.3 | 3.4 ± 0.4 | 0.16 |
|  | **BMI** (kg/m^2^) | 25.9 ± 4.2 | 25.0 ± 5.6 | 0.42 |

Data are expressed as mean±SD. BMI: Body Mass Index; Robust: CFS < 5; Frail: CFS >= 5
