# Supplementary material for: Prognostic Value of Glycated Hemoglobin in Frail Older Diabetic Patients With Hip Fracture
Source: Front Endocrinol (Lausanne). 2021 Nov 18;12:770400. doi: 10.3389/fendo.2021.770400 (PMC8637116; doi:10.3389/fendo.2021.770400)
Supplement: Supplementary file 3 [file Table_3.docx]

**Supplemental Table 3.** Antidiabetic drugs in frail and robust patients stratified by tertiles of HbA1c

|  | **Drug** | **Robust** (CFS < 5)  n = 37 | **Frail** (CFS >= 5)  n = 54 | p |
| --- | --- | --- | --- | --- |
|  | **Metformin** (%) | 23 (62.2) | 31 (57.4) | 0.65 |
|  | **Pioglitazone** (%) | 0 | 0 |  |
|  | **Sulfonylureas** (%) | 2 (5.4) | 2 (3.7) | 0.69 |
|  | **Repaglinide** (%) | 3 (8.3) | 4 (7.4) | 0.87 |
| **T1** (HbA1c < 48 mmol/mol) | **Acarbose** (%) | 0 | 1 (1.9) | 0.40 |
|  | **DPPIV-I** (%) | 8 (21.6) | 4 (7.4) | 0.05 |
|  | **GLP1R-a** (%) | 0 | 0 |  |
|  | **Gliflozins** (%) | 2 (5.4) | 1 (1.9) | 0.35 |
|  | **Rapid-acting insulin** (%) | 4 (10.8) | 7 (13) | 0.78 |
|  | **Long-acting insulin** (%) | 5 (13.5) | 5 (9.3) | 0.52 |

|  | **Drug** | **Robust** (CFS < 5)  n = 33 | **Frail** (CFS >= 5)  n = 31 | p |
| --- | --- | --- | --- | --- |
|  | **Metformin** (%) | 18 (54.5) | 20 (64.5) | 0.42 |
|  | **Pioglitazone** (%) | 0 | 1 (3.2) | 0.29 |
|  | **Sulfonylureas** (%) | 5 (15.2) | 4 (12.9) | 0.79 |
|  | **Repaglinide** (%) | 3 (9.1) | 4 (12.9) | 0.62 |
| **T2** (HbA1c 48-58 mmol/mol) | **Acarbose** (%) | 0 | 0 |  |
|  | **DPPIV-I** (%) | 5 (15.2) | 5 (16.1) | 0.91 |
|  | **GLP1R-a** (%) | 1 (3) | 1 (3.2) | 0.96 |
|  | **Gliflozins** (%) | 1 (3) | 0 | 0.33 |
|  | **Rapid-acting insulin** (%) | 4 (12.1) | 7 (22.6) | 0.27 |
|  | **Long-acting insulin** (%) | 4 (12.1) | 7 (22.6) | 0.27 |

|  | **Drug** | **Robust** (CFS < 5)  n = 15 | **Frail** (CFS >= 5)  n = 32 | p |
| --- | --- | --- | --- | --- |
|  | **Metformin** (%) | 10 (66.7) | 7 (21.9) | 0.003 |
|  | **Pioglitazone** (%) | 1 (6.7) | 0 | 0.14 |
|  | **Sulfonylureas** (%) | 3 (20) | 2 (6.3) | 0.15 |
|  | **Repaglinide** (%) | 1 (6.7) | 3 (9.4) | 0.76 |
| **T3** (HbA1c > 58 mmol/mol) | **Acarbose** (%) | 0 | 0 |  |
|  | **DPPIV-I** (%) | 2 (13.3) | 4 (12.5) | 0.94 |
|  | **GLP1R-a** (%) | 0 | 0 |  |
|  | **Gliflozins** (%) | 0 | 0 |  |
|  | **Rapid-acting insulin** (%) | 3 (20) | 13 (40.6) | 0.16 |
|  | **Long-acting insulin** (%) | 3 (20) | 17 (53.1) | 0.03 |

DPP-IV-I: Dipeptidyl peptidase-4 inhibitor; GLP1R-a: Glucagon-like peptide-1 receptor agonist
